# Supplementary material for: Activated astrocytes attenuate neocortical seizures in rodent models through driving Na+-K+-ATPase
Source: Nat Commun. 2022 Nov 21;13:7136. doi: 10.1038/s41467-022-34662-2 (PMC9681834; doi:10.1038/s41467-022-34662-2)
Supplement: Supplementary file 1 — Supplementary Information [file 41467_2022_34662_MOESM1_ESM.pdf]

Supplementary information for

Activated astrocytes attenuate neocortical seizures in rodent models through driving Na^+-K^+-ATPase

Junli Zhao[1,2,#], Jinyi Sun[2,#], Yang Zheng[1], Yanrong Zheng[1], Yuying Shao[2], Yulan Li[1], Fan Fei[1,2], Cenglin Xu[1], Xiuxiu Liu[2], Shuang Wang[3], Yeping Ruan[1], Jinggen Liu[1], Shumin Duan[4], Zhong Chen[1,2,3*] and Yi Wang[1,2,3*]

[1] Key Laboratory of Neuropharmacology and Translational Medicine of Zhejiang Province, School of Pharmaceutical Sciences, Zhejiang Chinese Medical University, Hangzhou, China;

[2] Institute of Pharmacology & Toxicology, College of Pharmaceutical Sciences, Zhejiang University, Hangzhou, China

[3] Epilepsy Center, Department of Neurology, Second Affiliated Hospital, School of Medicine, Zhejiang University, Hangzhou, China;

[4] Key Laboratory of Medical Neurobiology of the Ministry of Health of China, School of Brain Science and Brain Medicine, Zhejiang University, Hangzhou, China;

[#] These authors contributed equally to this paper.

***Correspondence to:**

Professor Zhong Chen, Ph.D., (ORCID, 0000-0003-4755-9357);

E-mail address: chenzhong@zju.edu.cn

Professor Yi Wang, Ph.D., (ORCID, 0000-0002-1350-2961);

E-mail address: wang-yi@zju.edu.cn

Tel & Fax: +86-571-86618083;

This PDF file includes:

Supplementary Figures 1-9

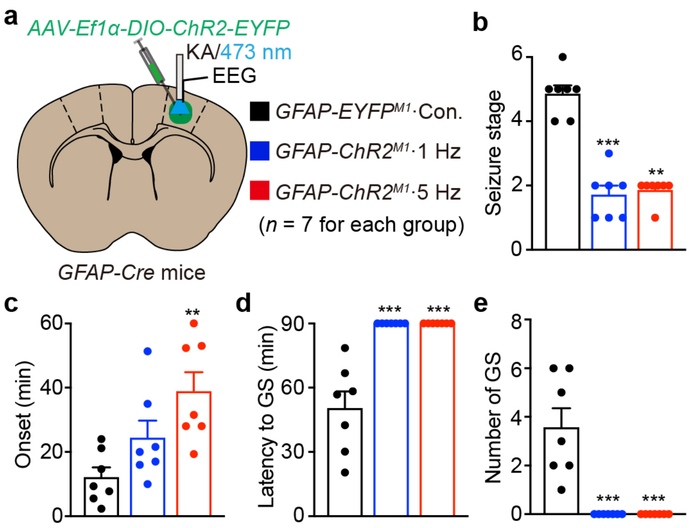

Supplementary Fig. 1: Optogenetic stimulation of ChR2-expressing astrocytes with different frequency attenuates KA-induced neocortical seizures.

a Schematic of viral injection, stimulation and EEG recording in *GFAP-ChR2*^{M1} mice. **b-e** Effects of different-frequency optogenetic stimulation of astrocytes on seizure stage (**b**), EEG onset (**c**), latency to GS (**d**) and number of GSs (**e**) in KA-induced seizures. $^{**}P < 0.01$, $^{***}P < 0.001$ compared with EYFP control group. Data shown as mean ± s.e.m. The number of mice used is indicated in figures. For detailed statistical information, see Supplementary Data 1. Source data are provided as a Source Data file.

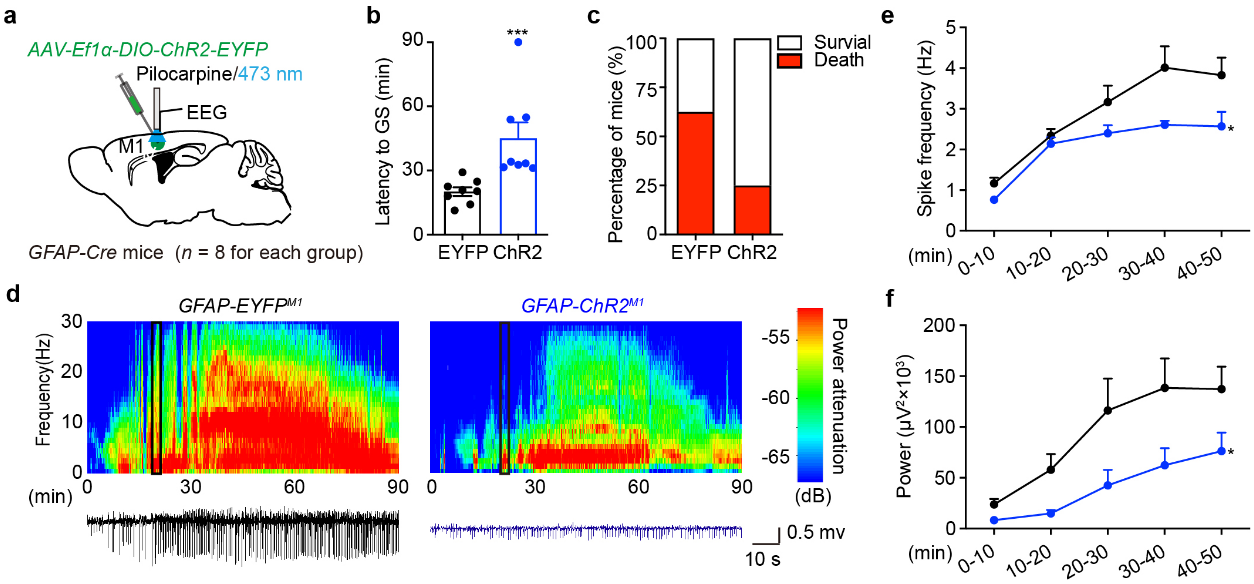

Supplementary Fig. 2：Optogenetic stimulation of ChR2-expressing astrocytes attenuates pilocarpine-induced neocortical seizures.

a Schematic of viral injection, blue light stimulation and EEG recording in *GFAP-ChR2*^{M1} mice. **b** Effect of optogenetic stimulation of astrocytes on latency to GS in pilocarpine-induced seizures. **c** Survival rate of mice in EYFP and ChR2-expressing groups. **d** Representative energy spectra and corresponding EEGs during seizure activity in pilocarpine-induced seizures. **e-f** Quantification of the average frequency of epileptic spikes (**e**) and EEG power (**f**) during seizure activity in EYFP and ChR2-expressing groups. $^{*}P < 0.05$, $^{***}P < 0.001$. Data shown as mean ± s.e.m. The number of mice used is indicated in figures. For detailed statistical information, see Supplementary Data 1. Source data are provided as a Source Data file.

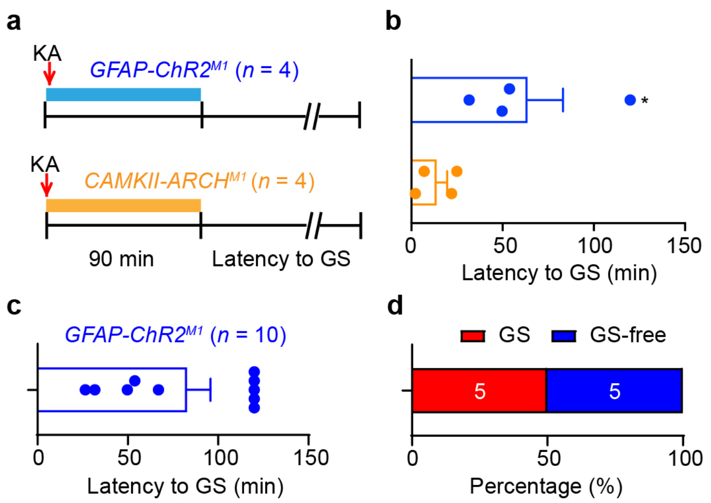

Supplementary Fig. 3：Optogenetic stimulation of ChR2 expressing-astrocyte attenuates seizures in a long-time manner.

a Latency to GS was recorded after blue/yellow light stimulation withdrawal in *GFAP-ChR2*^{M1} and *CAMKII-ARCH*^{M1} mice. **b** Comparation of the latency to GS in *GFAP-ChR2*^{M1} group and *CAMKII-ARCH*^{M1} group after light stimulation withdrawal. **c** Quantification of the latency to GS in *GFAP-ChR2*^{M1} group. **d** Percentage of GS-free in *GFAP-ChR2*^{M1} group after blue light withdraw. The cut off in this experiment is 120 min. If no GS occurred within 120 min, the latency was recorded as 120 min. $^{*}P < 0.05$ compared with *CAMKII-ARCH*^{M1} group. Data shown as mean ± s.e.m. The number of mice used is indicated in figures. For detailed statistical information, see Supplementary Data 1. Source data are provided as a Source Data file.

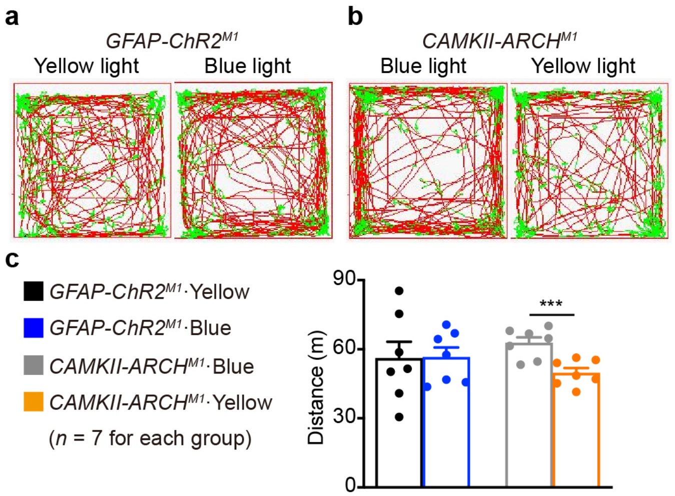

Supplementary Fig. 4: Optogenetic inhibition of pyramidal neurons impairs the motor function of mice.

a-b Representative yellow/blue light stimulation motion track of a *GFAP-ChR2*^{M1} mouse **(a)** and a *CAMKII-ARCH*^{M1} mouse (**b**). **c** Total distance traveled during 15 min in the open field test. $^{***}P < 0.001$ compared with control group. Data shown as mean ± s.e.m. The number of mice used is indicated in figures. For detailed statistical information, see Supplementary Data 1. Source data are provided as a Source Data file.

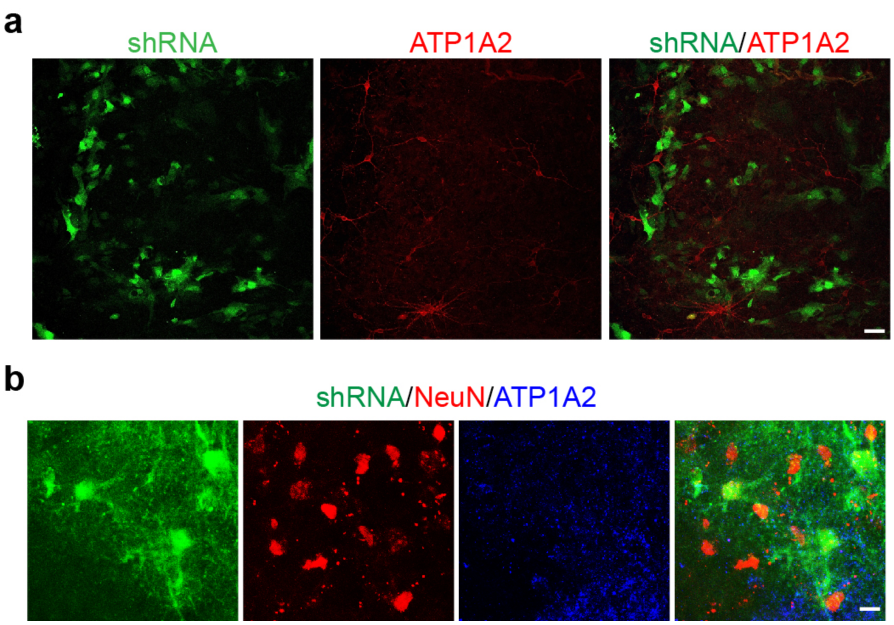

Supplementary Fig. 5：Validation of selective knockdown of Na^{+}-K^{+}-ATPase in astrocytes.

a Representative images of ATP1A2 expression in primary cortical cell culture, in the condition Na^{+}-K^{+}-ATPase inhibition by shRNA knockdown. Note that astrocytes transfected with GFAP-shRNA (Atp1α2)-GFP was no co-localized with ATP1A2 protein. **b** GFAP-shRNA (Atp1α2)-GFP was not co-localized with $NeuN^{+}$ neurons and has significantly reduced ATP1A2 expression in cortex.

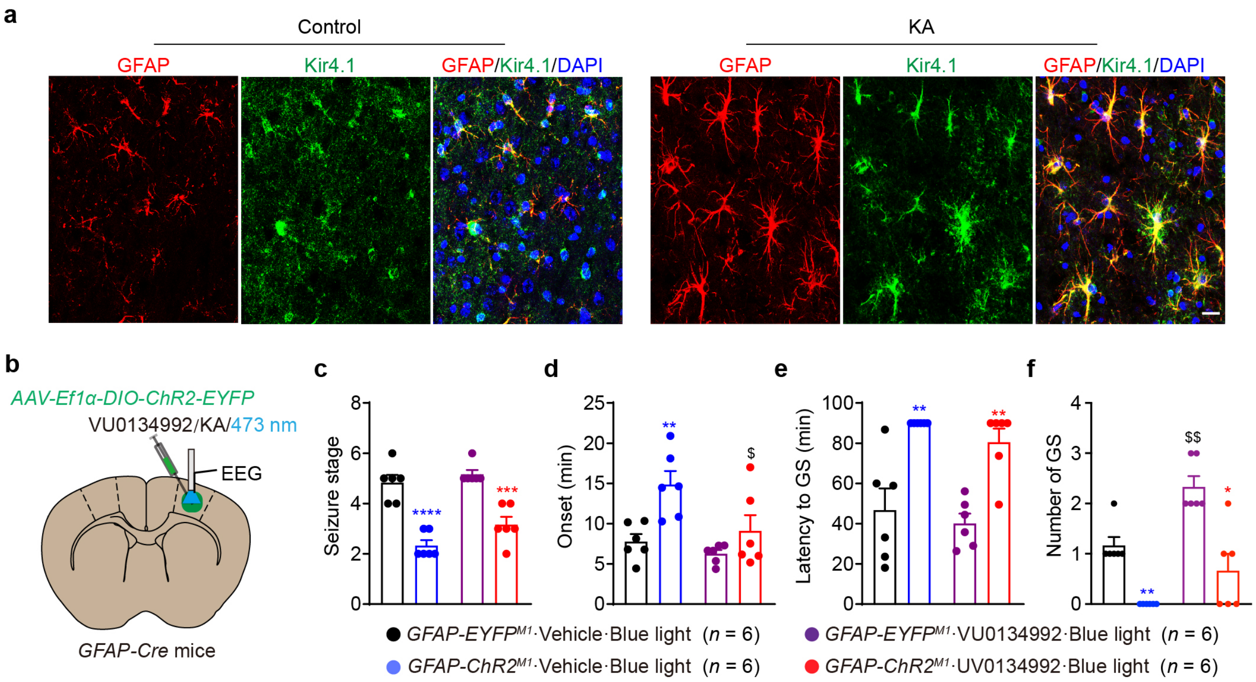

Supplementary Fig. 6: Astrocytic Kir4.1 is not required for the anti-seizure effect of optogenetic stimulation of astrocytes.

a Expression of Kir4.1 in control cortex and KA-induced seizure cortex. **b** Schematic of viral injection, blue light stimulation, drug injection and EEG recording in *GFAP-ChR2^{M1}* mice. **c-f** Effects of optogenetic stimulation of astrocytes on seizure stage (**c**), EEG onset (**d**), latency to GS (**e**) and number of GSs (**f**) in KA-induced seizures in the presence of Kir4.1 blocker VU0134992. $^{*}P < 0.05$, $^{**}P < 0.01$, $^{***}P < 0.001$, $^{****}P < 0.001$; $^{\$}P < 0.05$, $^{\$\$}P < 0.01$. Data shown as mean ± s.e.m. The number of mice used is indicated in figures. For detailed statistical information, see Supplementary Data 1. Source data are provided as a Source Data file.

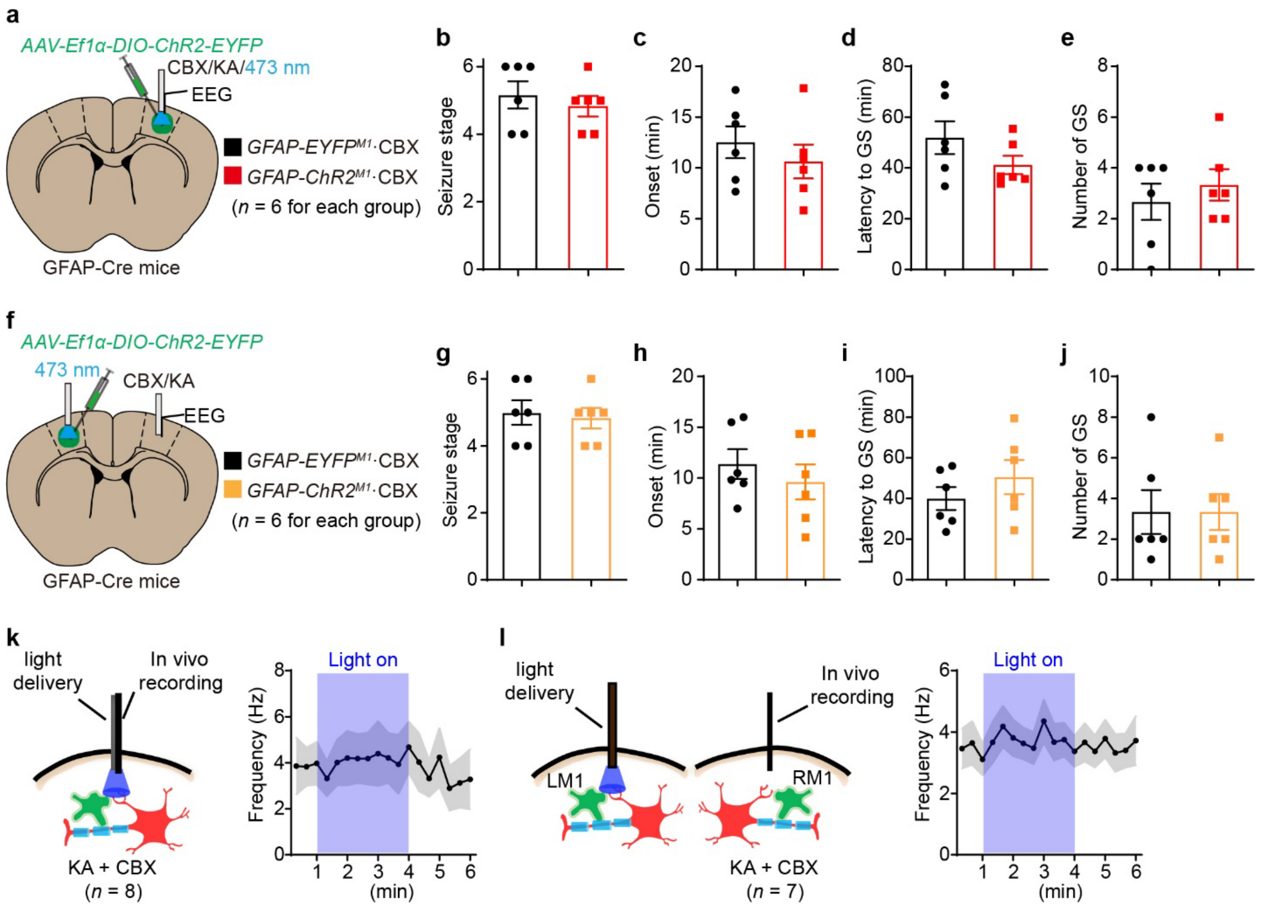

Supplementary Fig. 7: Astrocytic gap-junction inhibitor reverses the anti-seizure effect of optogenetic stimulation of astrocytes.

a Schematic of viral injection, blue light stimulation, drug injection and EEG recording in *GFAP-ChR2*^{M1} mice. **b-e** Effects of optogenetic stimulation of astrocytes on seizure stage (**b**), EEG onset (**c**), latency to GS (**d**) and number of GSs (**e**) in KA-induced seizures in the presence of gap junction inhibitor CBX. **f** Schematic of viral injection (LM1), stimulation (LM1) and EEG recording (RM1) in *GFAP-ChR2*^{M1} mice. **g-j** Effects of optogenetic stimulation of LM1 astrocytes on seizure stage (**g**), EEG onset (**h**), latency to GS (**i**) and number of GSs (**j**) after RM1 KA injection, in the presence of gap junction inhibitor CBX. **k, l** Schematic and time-frequency statistical charts of *in vivo* single-unit recording in *GFAP-ChR2*^{M1} mice in KA-induced seizure

status in the presence of gap junction inhibitor CBX with local (RM1, **k**) and remote (LM1, **l**) blue light stimulation. Data shown as mean ± s.e.m. The number of mice used is indicated in figures. For detailed statistical information, see Supplementary Data 1. Source data are provided as a Source Data file.

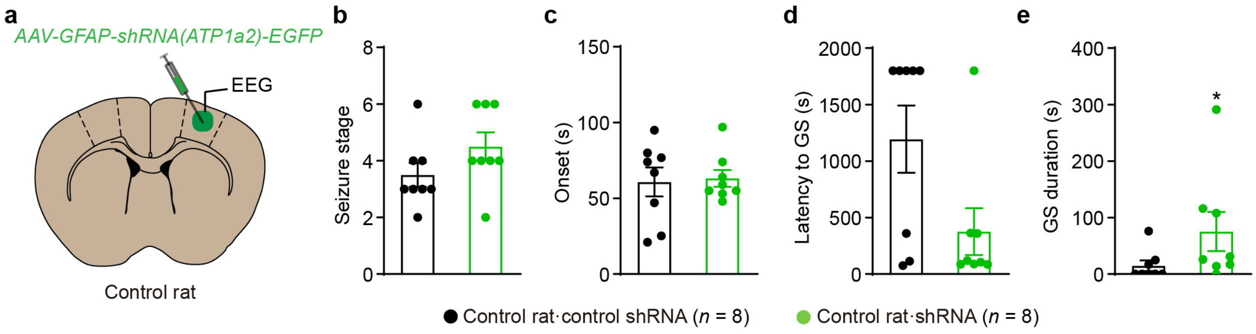

Supplementary Fig. 8: Astrocytic Na^+-K^+-ATPase inhibition in naive rats aggravates seizure severity.

a Schematic of viral injection and EEG recording in naive rat. **b-e** Effects of astrocytic Na^+-K^+-ATPase inhibition by shRNA knockdown on seizure stage (**b**), EEG onset (**c**), latency to GS (**d**) and GS duration (**e**) in PTZ-induced seizure model. $^*P < 0.05$ compared with control group. Data shown as mean ± s.e.m. The number of mice used is indicated in figures. For detailed statistical information, see Supplementary Data 1. Source data are provided as a Source Data file.

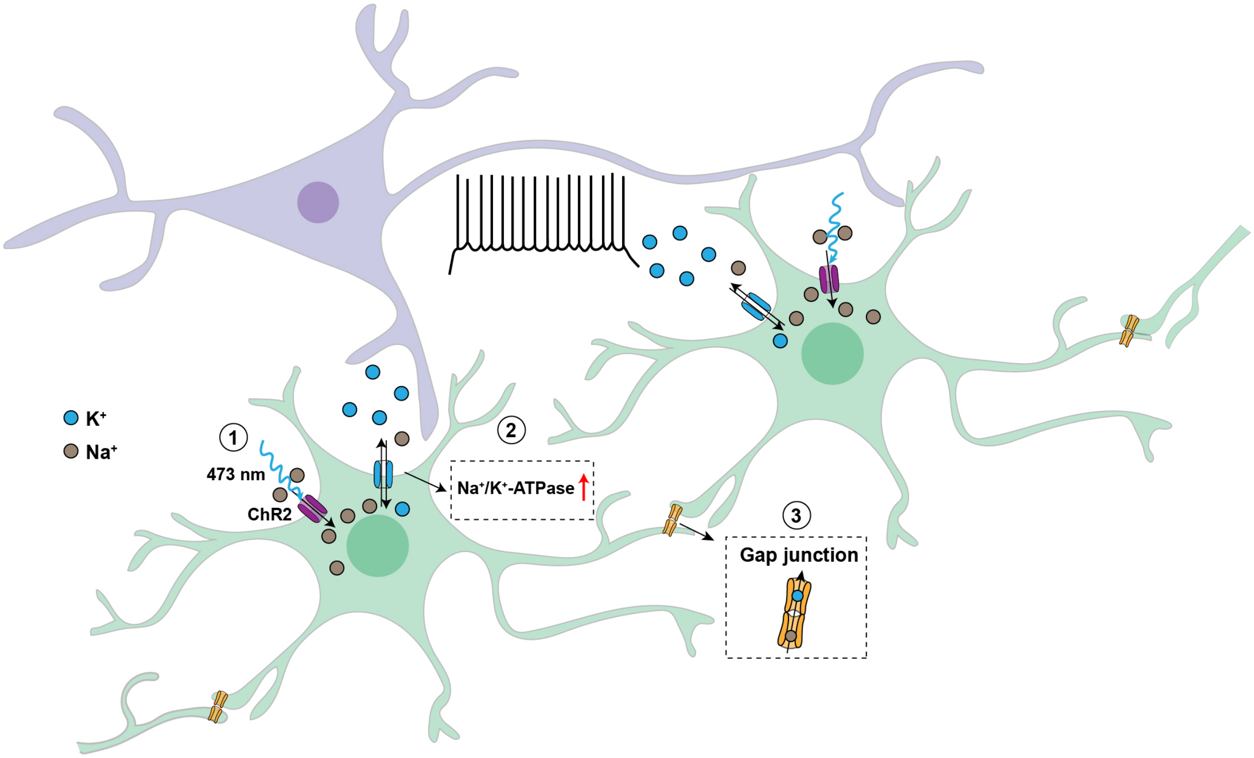

Supplementary Fig. 9：Summary of anti-seizure effect of optogenetic stimulation of astrocytes. 1) Blue light activates astrocytic ChR2 to induce the influx of Na^+ into astrocytes and then 2) activates the astrocytic Na^+-K^+-ATPase to buffer excessive K^+ surround neurons. 3) Gap junction between astrocytes contributes to spatial buffering of K^+ in the seizure condition.
