# Supplementary material for: Activated astrocytes attenuate neocortical seizures in rodent models through driving Na+-K+-ATPase
Source: Nat Commun. 2022 Nov 21;13:7136. doi: 10.1038/s41467-022-34662-2 (PMC9681834; doi:10.1038/s41467-022-34662-2)
Supplement: Supplementary file 3 — Description of Additional Supplementary Files [file 41467_2022_34662_MOESM3_ESM.docx]

Description of Additional Supplementary Files

**Legend for the Supplementary Data 1**

Statistical information for all figures in the manuscript
