# Supplementary material for: Activated astrocytes attenuate neocortical seizures in rodent models through driving Na+-K+-ATPase
Source: Nat Commun. 2022 Nov 21;13:7136. doi: 10.1038/s41467-022-34662-2 (PMC9681834; doi:10.1038/s41467-022-34662-2)
Supplement: Supplementary file 5 — Reporting Summary [file 41467_2022_34662_MOESM5_ESM.pdf]

Corresponding author(s): Zhong Chen; Yi Wang

Last updated by author(s): Oct 20, 2022

Reporting Summary

Nature Portfolio wishes to improve the reproducibility of the work that we publish. This form provides structure for consistency and transparency in reporting. For further information on Nature Portfolio policies, see our Editorial Policies and the Editorial Policy Checklist.

Statistics

For all statistical analyses, confirm that the following items are present in the figure legend, table legend, main text, or Methods section.

n/a	Confirmed	
☐	☒	The exact sample size (*n*) for each experimental group/condition, given as a discrete number and unit of measurement
☐	☒	A statement on whether measurements were taken from distinct samples or whether the same sample was measured repeatedly
☐	☒	The statistical test(s) used AND whether they are one- or two-sided<br>*Only common tests should be described solely by name; describe more complex techniques in the Methods section.*
☐	☒	A description of all covariates tested
☐	☒	A description of any assumptions or corrections, such as tests of normality and adjustment for multiple comparisons
☐	☒	A full description of the statistical parameters including central tendency (e.g. means) or other basic estimates (e.g. regression coefficient) AND variation (e.g. standard deviation) or associated estimates of uncertainty (e.g. confidence intervals)
☐	☒	For null hypothesis testing, the test statistic (e.g. *F*, *t*, *r*) with confidence intervals, effect sizes, degrees of freedom and *P* value noted<br>*Give P values as exact values whenever suitable.*
☒	☐	For Bayesian analysis, information on the choice of priors and Markov chain Monte Carlo settings
☒	☐	For hierarchical and complex designs, identification of the appropriate level for tests and full reporting of outcomes
☒	☐	Estimates of effect sizes (e.g. Cohen's *d*, Pearson's *r*), indicating how they were calculated

Our web collection on statistics for biologists contains articles on many of the points above.

Software and code

Policy information about availability of computer code

Data collection	PowerLab system with Bio Amplifiers (N12128, ADInstruments) was used for EEG recording.<br>ANYMZE 4.98 behavioral tracking software was used for locomotor studies.<br>Cerebus 6.04 system (Blackrock Microsystems) was used for in vivo single-unit recording.<br>Fiber photometry system (Thinkertech Nanjing Bioscience Inc, Co., Ltd.) was used for data collection.<br>Axon MultiClamp 700B Amplifier (Molecular Devices) was used for the acquisition of electrophysiological recordings.
Data analysis	LabChart 7 software was used for EEG analysis.<br>ANYMZE 4.98 software was used for open field data analysis.<br>Neuroexplorer 4.0. (Nex Technologies) software was used for in vivo single-unit recording data analysis.<br>Offline sort v4 software was used for in vivo single-unit data analysis.<br>MatLab software was used for fiber photometry data analysis.<br>Axon pClamp 10.3 software (Molecular Devices) was used for electrophysiological data analysis.<br>Prism GraphPad 8.0 (Graph Pad Software, Inc., USA) was used for statistical analysis.<br>Image-Pro Plus 7 software was used for imaging analysis.

For manuscripts utilizing custom algorithms or software that are central to the research but not yet described in published literature, software must be made available to editors and reviewers. We strongly encourage code deposition in a community repository (e.g. GitHub). See the Nature Portfolio guidelines for submitting code & software for further information.

Data

Policy information about availability of data

All manuscripts must include a data availability statement. This statement should provide the following information, where applicable:

- Accession codes, unique identifiers, or web links for publicly available datasets
- A description of any restrictions on data availability
- For clinical datasets or third party data, please ensure that the statement adheres to our policy

The authors declare that all data supporting the findings of this study are available within the paper and its supplementary information files "Source Data". The source data are provided as a Source Data file with the paper.

Field-specific reporting

Please select the one below that is the best fit for your research. If you are not sure, read the appropriate sections before making your selection.

[x] Life sciences   [ ] Behavioural & social sciences   [ ] Ecological, evolutionary & environmental sciences

For a reference copy of the document with all sections, see nature.com/documents/nr-reporting-summary-flat.pdf

Life sciences study design

All studies must disclose on these points even when the disclosure is negative.

Sample size	No statistical method was used to predetermine sample size. Sample sizes were estimated based on our previous studies for similar types of behavioral, biochemical, and electrophysiological analyses (PMID: 31987494, PMID: 28648501)
Data exclusions	Animals were excluded based of the lack of transgene expression in the targeted brain region.
Replication	All experiments were performed at least twice, including each treatment condition to prevent an unspecific day/condition effect. Each replicate experiment successfully recapitulated original findings.
Randomization	Allocation to experimental groups was done randomly.
Blinding	All experiments were conducted in a blind manner such that assays were conducted and analyzed without knowledge of the specific manipulation being performed.

Reporting for specific materials, systems and methods

We require information from authors about some types of materials, experimental systems and methods used in many studies. Here, indicate whether each material, system or method listed is relevant to your study. If you are not sure if a list item applies to your research, read the appropriate section before selecting a response.

Materials & experimental systems

n/a	Involved in the study	
[ ]	[x]	Antibodies
[x]	[ ]	Eukaryotic cell lines
[x]	[ ]	Palaeontology and archaeology
[ ]	[x]	Animals and other organisms
[x]	[ ]	Human research participants
[x]	[ ]	Clinical data
[x]	[ ]	Dual use research of concern

Methods

n/a	Involved in the study	
[x]	[ ]	ChIP-seq
[x]	[ ]	Flow cytometry
[x]	[ ]	MRI-based neuroimaging

Antibodies

Antibodies used	Primary antibodies: rabbit anti-GFAP antibody (rabbit, 1:400, BA0056, Boster), mouse anti-GFAP antibody (mouse, 1:400, G3893, Sigma), rabbit anti-NeuN antibody (rabbit, 1:400, MABN140, Millipore), mouse anti-ATP1A2 mAb (mouse,1:500, ab2871, Abcam), rabbit anti-ATP1A2 antibody (rabbit, 1:200, PA5-77512, Invitrogen), rat anti-GAPDH (rat, 1:5000; Kang-chen). Secondary antibodies: Alexa Fluor™ 488/594/647 secondary antibody (1:1000, Molecular Probes), goat anti-Rat/Mouse IgG (H + L) HRP (GRT007/GAM007, Multisciences).
Validation	1. rabbit anti-GFAP antibody: we used this antibody in our previous study (PMID: 28167116).<br>2. mouse anti-GFAP antibody: on the product website, it is indicated that this antibody has 1200 citations of papers that have used it. In the present study, we did not take additional steps to validate this antibody.

3. rabbit anti-NeuN antibody: we used this antibody in our previous study (PMID: 28167116).Additionally, on the product website, it is indicated that this antibody has 47 citations of papers that have used it. In the present study, we did not take additional steps to validate this antibody.
4. mouse/rabbit anti-ATP1A2 antibody: on the product website, this antibody has been used in at least 5 published papers. We did not take additional steps to validate this antibody.
5. rat anti-GAPDH antibody: we used this antibody in our previous studies (PMID: 28167116, PMID:31802434).

Animals and other organisms

Policy information about studies involving animals; ARRIVE guidelines recommended for reporting animal research

Laboratory animals	Adult (males, 2-4 months) GFAP-Cre mice (PMID: 28128211), CamkIIa-Cre mice (Jax No. 005359) and Sprague Dawley rats were used in this study. Two to five animals were housed in each cage on a 12-hour light/dark cycle with food and water ad libitum, and they were individually housed after surgery. The ambient temperature in animal facility was kept about 23–26 °C and humidity was about 50–60%. All behavior experiments were conducted between 9:00 and 17:00.
Wild animals	No wild animals were used in this study.
Field-collected samples	No field collected samples were used in this study.
Ethics oversight	All experimental procedures performed complied with the Zhejiang University Animal Experimentation Committee and were in complete compliance with the National Institutes of Health Guide for the Care and Use of Laboratory Animals.

Note that full information on the approval of the study protocol must also be provided in the manuscript.
