# Supplementary figures and images for: Activated astrocytes attenuate neocortical seizures in rodent models through driving Na+-K+-ATPase
Source: Nat Commun. 2022 Nov 21;13:7136. doi: 10.1038/s41467-022-34662-2 (PMC9681834; doi:10.1038/s41467-022-34662-2)

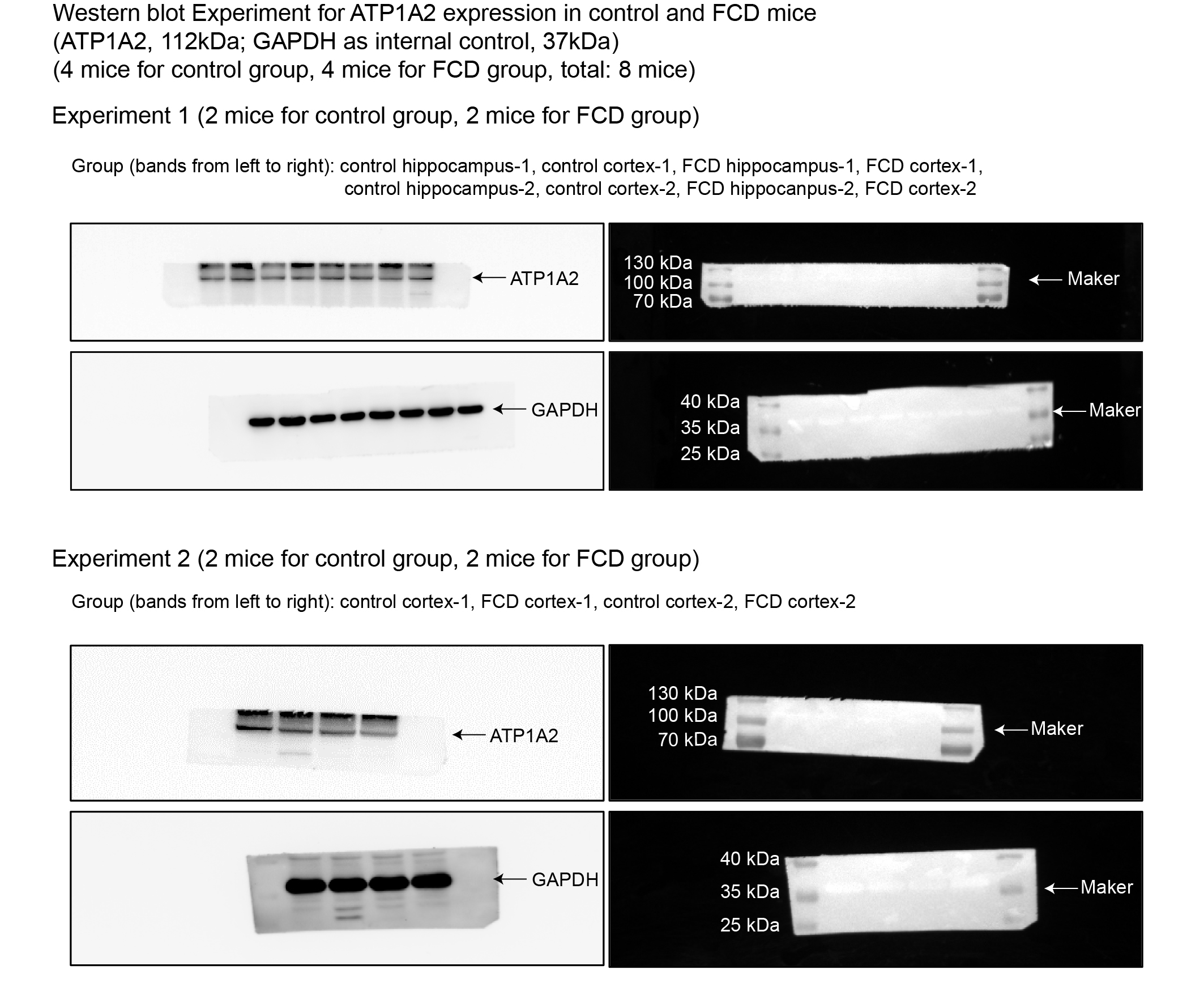

Supplement: Supplementary file 6 — Source Data [file 41467_2022_34662_MOESM6_ESM.zip › Source Data western blot.jpg]
